# *Camelina sativa* Methanolic and Ethanolic Extract Potential in Alleviating Oxidative Stress, Memory Deficits, and Affective Impairments in Stress Exposure-Based Irritable Bowel Syndrome Mouse Models

**DOI:** 10.1155/2020/9510305

**Published:** 2020-12-23

**Authors:** Roxana O. Cojocariu, Ioana-Miruna Balmus, Radu Lefter, Luminita Hritcu, Daniela C. Ababei, Alin Ciobica, Simona Copaci, Silvia E. L. Mot, Lucian Copolovici, Dana M. Copolovici, Stefana Jurcoane

**Affiliations:** ^1^Department of Research, Faculty of Biology, Alexandru Ioan Cuza University, B-dul Carol I, No. 11 Iasi, Romania; ^2^Department of Interdisciplinary Research in Science, Alexandru Ioan Cuza University of Iasi, Carol I Avenue, No. 11, Iasi 700506, Romania; ^3^Romanian Academy, Center of Biomedical Research, B-dul Carol I, No. 8 Iasi, Romania; ^4^Faculty of Veterinary Medicine, University of Agricultural Sciences and Veterinary Medicine “Ion Ionescu de la Brad” of Iasi, 3rd Mihail Sadoveanu Alley Iasi 700490, Romania; ^5^“Grigore T. Popa” University of Medicine and Pharmacy, Universitatii Street, No. 16, 700115 Iasi, Romania; ^6^Faculty of Biotechnology, University of Agronomic Sciences and Veterinary Medicine from Bucharest, Bd. Marasti, No. 59, 011464 Bucharest, Romania; ^7^Doctoral School of Biomedical Sciences, University of Oradea, Universității Str., No 1, 410087 Oradea, Romania; ^8^Faculty of Food Engineering, Tourism and Environmental Protection; Institute for Research, Development and Innovation in Technical and Natural Sciences, “Aurel Vlaicu” University of Arad, Elena Dragoi St. no 2, 310330 Arad, Romania; ^9^Academy of Romanian Scientists, Splaiul Independentei, No 54, Sector 5, 050094 Bucharest, Romania

## Abstract

*Camelina sativa* is mainly used as an oilseed crop; its edible oil is being also used as a traditional home remedy for the treatment of ulcers, wounds, and eye inflammations, due to the antioxidant activities. In the present study, the chemically characterized alcoholic extracts of *Camelina sativa* var. *Madalina* defatted seeds (5 g/kg body weight p.o., suspended in CMC-Na 0.1%) were administered to stress-induced animal models of irritable bowel syndrome (based on combinations of contention stress and multifactorial stress and maternal stress) and evaluated for the behavioural (short-term memory by the Y maze test, the anxious behaviour using the elevated plus maze test, and the antidepressant effect using the forced swimming test) and brain and bowel tissue oxidative status (superoxide dismutase and glutathione peroxidase enzymes activities and malondialdehyde and total soluble protein levels) improving effects. According to the chemical characterization, the extracts were rich in sinapine, glucosinolates, and flavonol glycosides. Moreover, this study showed the beneficial effects of *Camelina sativa* seed methanolic and ethanolic extracts on the behaviour and brain and bowel tissues oxidative stress status of stress exposure-based IBS mouse models. Despite the slight differences in the chemical composition of the methanolic and ethanolic extracts, the results suggested that the *Camelina sativa* extracts could reverse the short-term memory impairments caused by stress exposure and also could decrease the intensity and frequency of the anxiety and depressive-like behaviours observed in the stress-exposed animal models of IBS. Furthermore, the *Camelina sativa* extracts showed a significant effect on the oxidative stress markers in the brain and bowel tissues of the studied animal model by decreasing the superoxide dismutase activity and increasing the glutathione peroxidase activity. However, the results suggested that the extracts could also increase lipid peroxidation in bowel tissues. In this way, this study provides additional evidence that the administration of *Camelina sativa* seed alcoholic extracts could improve cognitive performances and mood and exhibit the antioxidant capacity in both the brain and bowel tissues.

## 1. Introduction

Irritable bowel syndrome (IBS) is currently characterized by ROME IV functional gastrointestinal diagnostic criteria as a brain-gut interaction disorder exhibiting digestive symptoms, such as abdominal pain, bloating, diarrhea and/or constipation, in the absence of any histological changes of the intestinal lining or biochemical alterations [1, 2]. The causes of IBS are not fully described yet, but they are considered to be complex and strongly influenced by individual variability and multifactoriality [3]. Thus, IBS pathogenesis is consisted in several essential components including genetic susceptibility, activation of the gut immune system, gastrointestinal infections, or dysregulation of the brain-gut axis [4, 5] in a complex interaction [6].

In this way, it was demonstrated that the bidirectional brain-gut axis disturbance could be involved in the abnormal function of the enteric or/and central nervous systems [7]. Considering that stress could potentially modulate the hypothalamic-pituitary-adrenal (HPA) axis activity, it could be suggested as a major IBS triggering factor addressing both the immune system and gastrointestinal tract functions [8, 9]. Also, the relationship between psychological stress and visceral hypersensitivity described by Musial et al. suggested that some mechanisms could be involved in visceral stimuli processing linked to the central and peripheral nervous systems [10]. In this context, the bidirectional brain-gut axis impairment could be a very effective model of IBS pathophysiology in animals.

Moreover, the brain–gut impairments accompanied by functional gastrointestinal symptoms were described in several neuropsychiatric disorders, such as depression and anxiety [11, 12]. In this context, we previously demonstrated that chronic restraint stress could lead to anxiety- and depressive-like behaviours which were in significant correlation to brain oxidative status changes [13, 14]. Due to the previously described implications of the chronic stress exposure on the HPA axis modulation and also on the mood spectrum symptoms occurrence, it is now considered that the most efficient functional gastrointestinal disorder animal models are based on stress exposure [13]. Moreover, it was demonstrated that chronic stress exposure could alter neuroendocrine, neurochemical, and sensory response to nociceptive stimuli along the brain-gut axis [15] and faecal microbiota [16] and induce visceral hypersensitivity. Furthermore, maternal separation as well as chronic water avoidance and acute restraint stress could lead to increased colonic motility and visceral hyperalgesia in rats [17–19].

Regarding the oxidative stress implications in IBS, we previously described several correlations between the IBS pathological mechanisms and oxidative stress occurrence [20] and also recent reports showed that both signalling impairments and oxidative balance changes occur in some gastrointestinal and neurological disorders [21, 22]. In this way, the oxidative changes were described in both IBS patients [23–25] and animal models [26, 27].

*Camelina sativa* is a flowering plant in the *Brassicaceae* family, native to northern Europe and central Asia, traditionally cultivated as an oilseed biofuel crop to produce vegetable oil and animal feed [28, 29]. The oil extracted from seeds is rich in linolenic acid (C18:3*ω*3, ALA), an essential *ω*3 fatty acid (*ω*3FA), whose consumption has been associated with a decreased risk of coronary heart disease and inflammatory diseases [30–38] and some beneficial effects on behavioural manifestations and oxidative stress parameters in a zymosan-induced IBS mouse model [39]. Most of the therapeutical properties of the *Camelina sativa* seeds are due to phenolic content, such as sinapine and phytic acid [40], antioxidants (tocopherols) [29], flavanols (quercetin) [41], and glucosinolates [42].

In this context, to our best knowledge, no previous study described the potential of *Camelina sativa* seed alcoholic extracts in alleviating the behavioural and oxidative burden of chronic stress. Therefore, the aim of this study was to describe the cognitive and antioxidant effects of two *Camelina sativa* seed extracts (methanolic and ethanolic) in a complex mouse model of IBS, based on chronic stress exposure. Consequently, we obtained two alcoholic extracts from an indigenous *Camelina sativa* variety (var. *Madalina*) defatted seeds and evaluated their effect on the cognitive performances (short-term memory in the Y maze test), anxiety-like behaviour changes (in the elevated plus maze test), and depressive-like behaviour occurrence (in the forced swimming test), in a complex chronic stress-based irritable bowel syndrome mouse model by comparison to double negative and positive controls. Furthermore, the brain and bowel tissues of the animals were subjected to biochemical assessment of oxidative stress status (superoxide dismutase and glutathione peroxidase enzyme activities and malondialdehyde and total soluble protein content).

## 2. Materials and Methods

### 2.1. *Camelina sativa* Seed Extract Preparation

*Camelina sativa* var. *Madalina* was cultivated in natural conditions (without using any fertilizers) at Moara Domnească Teaching Farm, University of Agronomic Sciences and Veterinary Medicine, Bucharest. Following harvesting, the seeds were grounded by using a coffee bean grinder (ZASS, Germany) until a fine powder was obtained. 10 g of fine *Camelina sativa* seed powder was defatted with 80 ml n-hexane (Gerhardt Soxtherm, Multistat 402, Germany) for two hours and left for drying in the chemical hood. For this study, the methanolic extract (ME) was obtained by processing 5 g fine seed powder together with 20 ml aqueous solution MeOH (90%) by 1 minute vortexing and two days gentle shaking (microplate shaker, VWR, USA) in the dark. The ethanolic extract (EE) was also obtained from 5 g of defatted seed powder that were vortexed with 20 ml EtOH aqueous solution (90%) for 1 min and then gently shook in the dark for two days. Following centrifugation (Hettich, Rotina 380R, Germany), the supernatants of both the alcoholic extracts were concentrated by a rotary evaporator (IKA RV10, Germany). The extracts were kept at +4°C for further analyses. Adequate dilutions of the extract samples were prepared for the chromatographic analyses and *in vivo* experiments.

### 2.2. Extract Chemical Composition Determination

The chemical composition of the alcoholic extracts of *Camelina sativa* var. *Madalina* was determined by using a high-performance liquid chromatograph coupled with a diode array detector and a mass spectrometer detector (UHPLC-DAD-MS: UHPLC: Nexera X2, DAD model M30A, MS Model 8040, Shimadzu, Japan). A NUCLEOSIL HPLC C-18 reversed-phase column (EC 100/4.6 NUCLEOSIL 100-3 C18, 100 × 4.6 mm, particle size: 3.0 *μ*m, length: 10 cm, MACHEREY-NAGEL GmbH & Co. KG, Germany) was employed for chromatographic separation. A modified method of separation of the compounds of an earlier reported method was used [43]. In the present study, the composition of the mobile phase of the gradient elution was as follows: (A) distilled water with 0.1% trifluoroacetic acid and (B) acetonitrile with 0.1% trifluoroacetic acid. The gradient was applied at a flow rate of 0.5 ml/min, and the binary gradient with linear interpolation was used as follows: 0 min, 5% B; 5 min, 42% B; 25 min, 35%B; and 5 min, 5% B. The column and samples were incubated at +25°C. The injection volume of the sample was 10 *μ*l. The mass spectra were recorded using both positive and negative ionisation modes, respectively. The dry nitrogen was heated to 250°C, and the drying gas flow was 15 l/min. Data were acquired in the positive and negative scan modes in the range of 100–1200 Da. The compounds were identified by their UV-VIS spectra ranging from 190 to 600 nm, by comparing their retention times with standards and by analysing their recorded mass spectra.

### 2.3. Animals

Male Swiss mice weighing 30-40 g were housed in polyacrylic cages containing woodchip bedding (5 animals/cage, woodchips changed periodically) and habituated in constant environmental conditions (20°C, 55-60% humidity, natural light-dark cycle, and free access to water and food). Animal care and experimental procedures were conducted according to the regulations regarding animal use in biomedical research. The study was approved by the Ethics Committee of USAMV (no. 385/04.04.2019), and all efforts were made to reduce the number of tested animals and their suffering.

### 2.4. Experimental Design

Thirty-six neonatal male mice (*n* = 36) were subjected to maternal separation between postnatal days (PD) 1 and 14 and to 3 days contention stress between postnatal days PD 90 and PD 92. Between PD 93 and PD 98, they were subjected to chronic unpredictable mild stressors exposure as follows: (1) exposure to sound predator (birds of prey cries lasting 10 minutes at ambient level), (2) water deprivation, (3) injection simulation, (4) tilt cages backward at 45 degrees during 1 to 4 hours, (5) 1-minute tail pinch at 1 cm from the end of the tail, and (6) 24 hours food deprivation. Concomitantly, the mice were subjected to water avoidance stress. The control group was subjected to identical environmental conditions in the absence of any studied stress factors.

**Figure 1:**
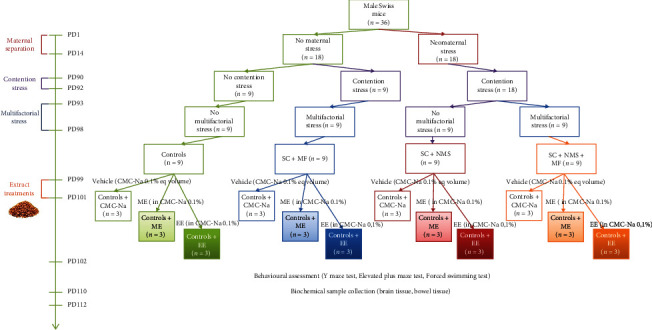
The schematic description of the experimental design and timeline.

Given the described stress paradigms, the animals were divided into four groups: (1) control, (2) contention stress+neomaternal separation+multifactorial stress-exposed group (SC+NMS+MF), (3) contention stress+multifactorial stress-exposed group (SC+MF), and (4) contention stress+neomaternal separation (SC+NMS). Each group was divided into three subgroups, depending on the subsequent treatment (vehicle solution–CMC-Na 0.1% and methanolic and ethanolic extracts of *Camelina sativa* seeds) (Figure 1).

### 2.5. *Camelina sativa* Extract Administration

The extracts were suspended in CMC-Na 0.1% at volume which could facilitate proper administration (maximum 0.5 ml suspension per administration) and meeting the 5 g/kg body weight dose. Between PD 99 and PD 101, the CMC-Na+extract suspensions were orally administered to the mice using a gavage needle, according to the standard procedures and protocols. The control animals also received orally the same CMC-Na volume to facilitate similar hydration conditions for all the animals.

### 2.6. Behavioural Testing

Behavioural assessment was conducted following the stress exposure and extract administration between PD 102 and PD 110 in this order: Y maze test, elevated plus maze test, and forced swim test.

### 2.7. Y Maze Test

Short-term memory performance was evaluated using the Y maze test by assessing the successive and alternating exploratory behaviour in the three arms of the Y-shaped apparatus (40 cm length, 8 cm width, and 15 cm height, attached at 120-degree angles) [44]. The maze was cleaned with alcohol-free disinfectant wipes between each trial. The spontaneous alternation behaviour was defined as the consecutive entries in all three arms of the maze and was calculated as percentage of actual alternations per maximum alternations (total number of arms entered minus 2). An arm entry was counted when the hind paws of the mouse were entirely within the arm.

### 2.8. Elevated plus Maze

The elevated plus maze test (EPM) was administered according to the protocol described by the Pellow group [45] using a mouse dimensioned maze. The maze consisted in four arms arranged to form a plus profile. Two opposite arms were enclosed by vertical opaque walls, while the other two were opened. Following the transfer to the testing room, the animals were placed in the middle of the maze facing the closed arms for a 5-minute exploring session. The time spent in the opened and closed arms and the number of entries in the arms were recorded. An arm entry was counted when the hind paws of the mouse were entirely within the arm. Also, the grooming behaviour was recorded considering its anxiety-related behavioural relevance. The parameters relevant to the behavioural assessment carried by this test were calculated as % time spent in the opened arms (opened arms time/total arms time × 100) and % opened arms entries (open arms entries/total arms entries × 100).

### 2.9. Forced Swim Test

Behavioural despair was assessed using Porsolt's forced swim test (FST) [46]. The protocol consists of evaluating swimming behaviour while maintaining the individuals in a transparent glass cylinder (30 cm in diameter, height 59 cm) filled with water (25 cm, 26°C) from which the animal could not escape. The animals were exposed to the experimental conditions for 15 minutes on the first day of accommodation and learning. Following this training session, the animals were safely removed from the water cylinder, dried using absorbent textile material, and returned to their habitant cages. On the following day, the animals were subjected to a similar procedure (6-minute test session) in which the duration of their behaviour was recorded: mobility, floating (minimal movement to maintain the head above the water level), and struggle (similar to climbing movement, using the forearms trying to escalade the glass cylinder).

### 2.10. Tissue Collection and Sample Preparation

Following behavioural testing, during the PD 112, biological samples of the brain and bowel tissues were collected in total anaesthesia conditions (ketamine 100 mg/kg, xylazine 10 mg/kg). The bowel tissues were washed with autoclaved purified water before further processing. Brain and colon extracts were prepared using tissue extraction buffer (0.328 g TRIS, 1.304 g KCl_2_, and distilled water to 200 ml volume, pH = 7.4) at 1 : 10 *w*/*v* ratio and kept at -22°C until biochemical assessment.

### 2.11. Biochemical Determinations

Superoxide dismutase (SOD) activity was measured using 19160 SOD Assay Kit (Sigma Aldrich, Germany) according to the manufacturer's instruction. The determination is based on the percentage of reaction inhibition rate of enzyme with WST-1 substrate (a water-soluble tetrazolium dye) and xanthine oxidase according to the manufacturer's instructions. Each endpoint assay was monitored by absorbance at 450 nm (the absorbance wavelength for the coloured product of WST-1 reaction with superoxide) after 20 min of reaction time at 37°C.

Glutathione peroxidase (GPx) activity was measured using the GPx Cellular Activity Assay Kit CGP-1 (Sigma Chemicals). This indirect method is based on the oxidation of glutathione (GSH) to oxidized glutathione (GSSG) which is catalysed by GPx and coupled with recycling GSSG to GSH by glutathione reductase (GR) in the presence of NADPH. The decrease in NADPH at 340 nm during oxidation to NADP^+^ is an indicator of GPx activity. The antioxidant enzyme activities were normalized by total soluble protein content assessed using Bradford assay [47].

Malondialdehyde (MDA) levels were determined by thiobarbituric acid reactive substance (TBAR) assay. 200 *μ*l of supernatant was added and mixed with 1 ml of 50% trichloroacetic acid, 0.9 ml of Tris–HCl (pH 7.4), and 1 ml of thiobarbituric acid 0.73%. Following vortexing, the samples were maintained in a water bath at 100°C for 20 min. Afterwards, the samples were centrifuged at 3000 rpm for 10 min and supernatant read at 532 nm. The signal was read against an MDA standard curve and normalized against total soluble protein content.

### 2.12. Statistical Analysis

The numerical data obtained in behavioural and biochemical evaluation were statistically analysed using one-way analysis of variance (ANOVA), Grubbs's test for outliers, and Fisher's LSD test using Minitab 19 (State College, Pennsylvania, USA, 2019). The results are expressed as means ± SEM and were regarded statistically significant at *p* < 0.05. Statistical correlations were expressed as Pearson's linearity coefficient.

## 3. Results

### 3.1. Chemical Characterization of the Alcoholic Extracts

The chemical characterization of the extracts obtained from defatted *Camelina sativa* var. *Madalina* seeds could vary depending on the solvent used for extraction. Ten compounds were obtained in the ethanolic extract (EE) and eight compounds in the methanolic extract (ME), as depicted in Table 1. The compounds were identified by using their UV-VIS spectra compared with the authentic standards and the recorded mass spectra and also by comparison with the previous reports in scientific literature.

The major compound found in the extracts is sinapine (C_16_H_24_NO_5_, Mw = 310.365 Da, m/z: 310, 230, 216, 172) with 51.572% and 31.681% relative amount in methanolic and ethanolic extracts, respectively. Also, three glucosinolates were identified: glucoarabin (9-methylsulfinylnonyl glucosinolate, C_17_H_33_NO_10_S_3_, Mw = 507.126 Da, m/z found: 506 Da in negative mode), glucocamelinin (10-methylsulfinyldecylglucosinolate, C_18_H_35_NO_10_S_3_, Mw = 521.142 Da, m/z found: 520 Da), and gluconesliapaniculatin (11-methylsulfinylundecylglucosinolate, C_19_H_36_NO_10_S_3_, Mw = 534.15 Da, m/z found: 534 Da). Moreover, two flavanol glycosides were observed: quercetin-2^″^-O-apiosyl-3-O-rutinoside (C_32_H_37_O_20_, Mw = 741.187 Da, m/z found: 641 Da) and rutoside (quercetin-3-O-rutinoside or rutin, C_27_H_30_O_16_, Mw = 610.518 Da, m/z found: 611 Da). Five other compounds were not identified (Table 1).

### 3.2. The Effect of *Camelina sativa* var. *Madalina* Seed Extracts on Animal Behaviour

#### 3.2.1. The Effect of *Camelina sativa* Methanolic and Ethanolic Extracts on Short-Term Memory

**Figure 2:**
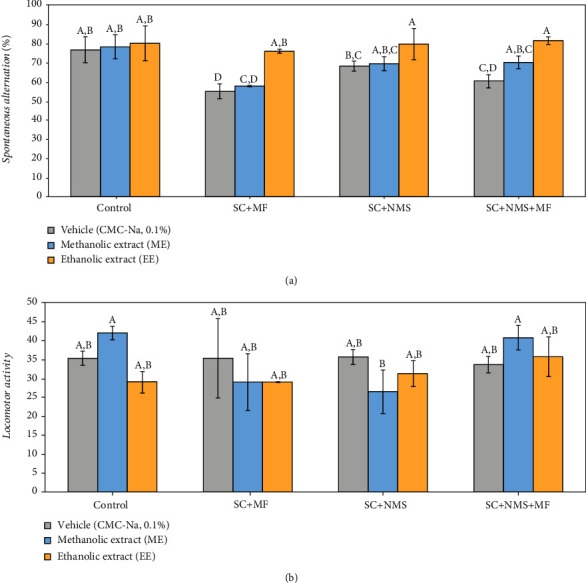
The effects of *Camelina sativa* var. *Madalina* seed methanolic (ME) and ethanolic (EE) extracts in complex irritable bowel syndrome mouse models on the short-term spatial memory (a) and locomotor activity (b), as observed in the Y maze test. The values are expressed as mean ± S.E.M (*n* = 3 per group; C: control; SC: contention stress; NMS: maternal separation; MF: multifactorial stress; ME: methanolic extract; EE: ethanolic extract; A, B, C, D: Fisher's LSD test).

Regarding the performance of mice in the Y maze task, although we did not obtain statistically significant differences between the stress-exposed groups to which the methanolic or ethanolic extract was administered, we observed a significant overall effect in terms of spontaneous alternation regarding short-term spatial memory (*F* (11, 24) = 3.89, *p* = 0.000874), but no significant differences in terms of locomotor activity (*F* (11, 24) = 1.18, *p* = 0.331]. Further analysis of the data showed no significant variations between the nonstressed control group which received the methanolic extract (*p* = 0.819) or the nonstressed control group which received the ethanolic extract (*p* = 0.646), as compared to the control group (C) (Figure 2(a)). Also, when we compared the effect of the two extracts in the stress-exposed groups, respectively, controls, we observed no statistical differences or variations between the control+EE and control+ME groups (*p* = 0.817).

The improvement of short-term memory was observed in the multifactorial and contention stress-exposed group which received EE, as compared to ME (*p* = 0.018) and saline (*p* = 0.007). Similarly, the EE was shown to be more effective in improving short-term memory in the joint stress-exposed mice (SC+MF+NMS), as compared to ME (*p* = 0.05). Also, a significant increase in short-term memory performance was observed in contention stress-exposed maternally separated mice while receiving EE (*p* = 0.048, as compared to stressed nontreated corresponding group), and no important difference between the effect of ME and EE on this stress type (*p* = 0.116). However, it was observed that short-term memory performance was still significantly decreased in the ME-treated stressed mice as compared to ME positive control (*p* = 0.008).

#### 3.2.2. The Effect of *Camelina sativa* Methanolic and Ethanolic Extracts on the Anxiety-Like Behaviour

**Figure 3:**
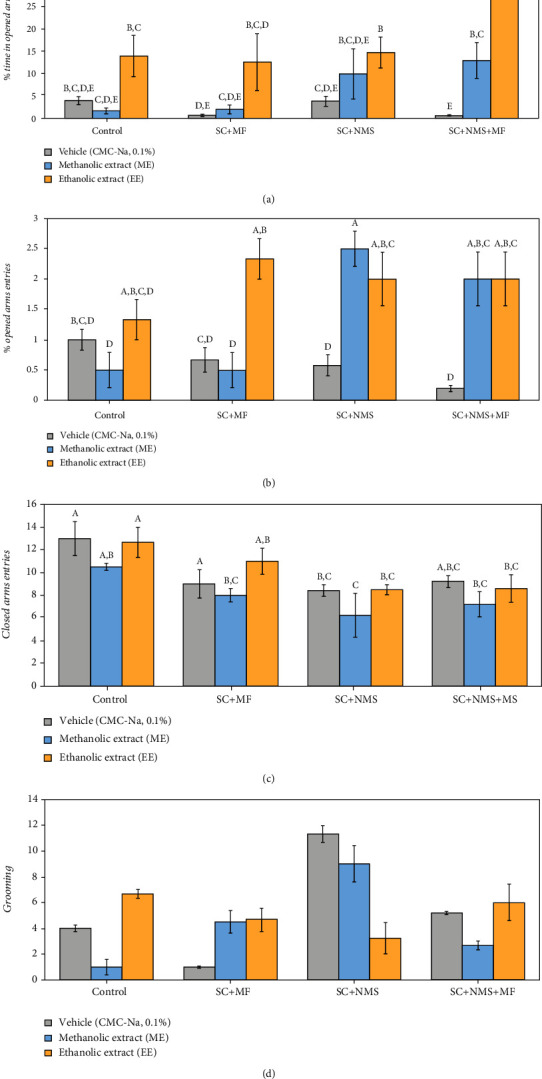
The effects of *Camelina sativa* var. *Madalina* seed methanolic (ME) and ethanolic (EE) extracts in a complex irritable bowel syndrome mouse model on the anxious-like behaviour, as observed in the elevated plus maze test: (a) open arms time (%), (b) open arms entries (%), (c) closed arms entries, and (d) grooming. The values are mean ± S.E.M (*n* = 3 per group). C: control; SC: contention stress; NMS: neomaternal separation; MF: multifactorial stress; A, B, C, D: Fisher's LSD test grouping).

Anxiety-like behaviour was assessed in the elevated plus maze test, and we found a significant group difference in terms of open arms time (OAT) (*F* (11, 24) = 6.04, *p* = 1.5*E*^−05^) and open arms entries (OAE) (*F* (11, 24) = 2.05, *p* = 0.0045). Further analysis showed no difference in terms of anxious behaviour occurrence in the nonstressed groups while receiving ME (*p* = 0.719) and EE (*p* = 0.128), but significant changes in mobility while administered to contention and multifactorial stress-exposed mice (OAE in SC+MF-ME vs. EE, *p* = 0.026) (Figure 3(a)).

Significant anxiolytic effect was observed in joint stress-exposed group (SC+MF+NMS) receiving ME and EE (*p* < 0.001) (Figures 3(a) and 3(b)), but with a greater yield for EE (*p* = 0.01, as compared with ME) (Figure 3(b)). Significant improving effects of EE were observed in anxiety-like behaviour in the contention stress-exposed maternally separated mice (*p* = 0.034) and only minor improving effects of the same extract were observed in the contention and multifactorial stress-exposed mice (*p* = 0.07) (as observed by evaluating the behavioural parameter of open arms time, Figure 3(b)). However, the significant improvement observed for both extracts administered to the joint stress-exposed mice (*p*_ME_ = 0.013, *p*_EE_ < 0.001), the anxiety-like behaviour was still significantly increased for this groups, as compared to the positive control (*p*_ME_ = 0.05, *p*_EE_ = 0.014, versus C+extract groups, respectively).

As it is also a significant parameter to evaluate the anxious behaviour, the closed arms entries index did not vary among the experimental groups (*F* (11, 24) = 2.03, *p* = 0.052) (Figure 3(c)). Despite that, we observed that both ME (*p* = 0.045) and EE (*p* = 0.049) could exhibit the anxiolytic effect when administered in contention stress-exposed maternally separated mice, as compared to positive controls, respectively. Similarly, we observed that the negative effects of stress exposure on affective status are significantly alleviated by the *Camelina sativa* var. *Madalina* seeds extract in the contention and multifactorial stress-exposed mice (*p*_EE_ = 0.041), contention stress-exposed maternally separated mice (*p*_ME_ = 0.003, *p*_EE_ = 0.024), and joint stress-exposed mice (*p*_ME_ = 0.004), despite that there are also significantly different from the extract positive controls (*p*_ME_ = 0.04). By comparison, we observed that the methanolic extract was more effective in alleviating anxious behaviour in contention stress maternally separated mice (*p* = 0.01) and joint stress exposure mice (*p* = 0.004), as compared to contention and multifactorial stress mice. Regarding the anxiolytic behaviour occurrence (grooming), we observed no significant overall difference between groups (*F* (11, 24) = 0.26, *p* = 0.988) (Figure 3(d)).

#### 3.2.3. The Effect of *Camelina sativa* Methanolic and Ethanolic Extracts on the Depressive-Like Behaviour

**Figure 4:**
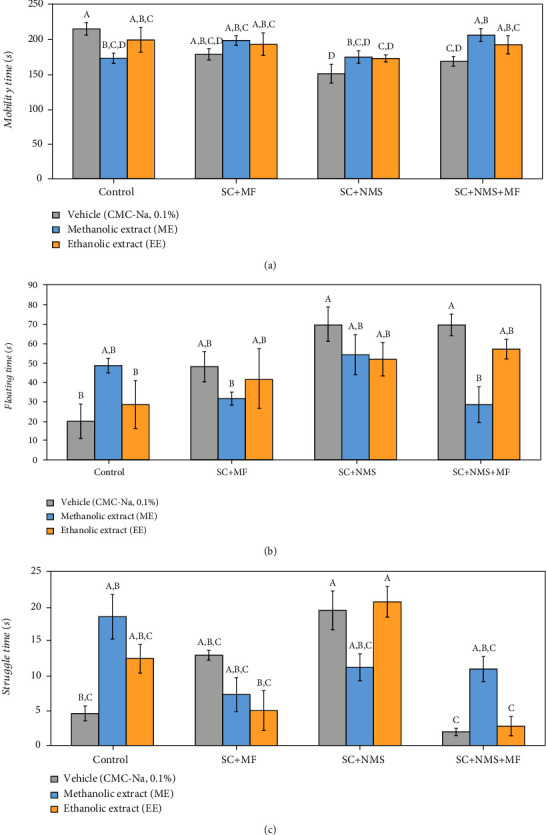
The effects of *Camelina sativa* var. *Madalina* methanolic (ME) and ethanolic (EE) extracts in complex irritable bowel syndrome mouse model on the depressive-like behaviour in the forced swim test: (a) mobility time (s), (b) floating time (s), and (c) struggle time (s). The values are mean ± S.E.M (*n* = 3 per group). C: control; SC: contention stress; NMS: neomaternal separation; MF: multifactorial stress; A, B, C, D: Fisher's LSD test grouping).

While evaluating the effects of the two studied seed extracts on the depressive-like behaviour occurrence in the complex irritable bowel syndrome mouse model, we observed significant overall changes on mobility time (*F* (11, 24) = 2.96, *p* = 0.006). Moreover, we observed that there are significant differences between the two extracts while administered to nonstressed mice (*p* = 0.031), while EE was considered less efficient in decreasing the depressive-like behaviour occurrence (*p* = 0.393). Furthermore, the positive effect of ME in regarding the antidepressant potential was observed in contention stress maternally exposed mice (*p* = 0.011) and joint stress-exposed mice (*p* = 0.05) (Figure 4(a)). No significant overall difference between the studied groups were obtained for the floating time parameter (*F* (11, 24) = 2.34, *p* = 0.27). However, we observed a significant antidepressant effect of ME in joint stress-exposed mice (*p* = 0.002), as compared to positive control (Figure 4(b)).

Regarding the struggling behaviour, we observed a significant overall difference between the studied groups (*F* (11, 24) = 2.10, *p* = 0.045). In this matter, we observed that the EE had a better yielding in decreasing the depressive-like behaviour occurrence in contention and multifactorial stress exposure mice (*p* = 0.049) and joint stress exposure mice (*p* = 0.013), as compared to contention stress-exposed maternally separated mice (Figure 4(c)).

### 3.3. The Effect of *Camelina sativa* var. *Madalina* Seed Methanolic and Ethanolic Extracts on Oxidative Status Markers

#### 3.3.1. The Effect of *Camelina sativa* Seed Methanolic and Ethanolic Extracts on Brain Tissue Oxidative Stress

**Figure 5:**
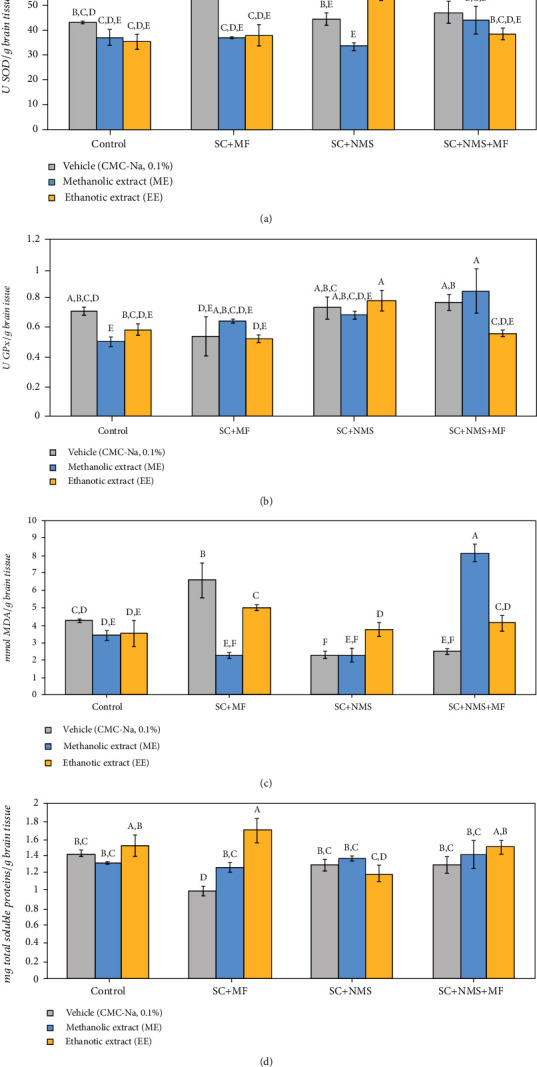
The effects of *Camelina sativa* var. *Madalina* seed methanolic (ME) and ethanolic (EE) extracts on brain oxidative stress markers in complex irritable bowel syndrome mouse model. (a) SOD-specific activity (U/g brain tissue). b) GPx-specific activity (U/g brain tissue). (c) MDA brain content (mmol MDA/g brain tissue). (d) Total soluble proteins brain content (mg proteins/g brain tissue). The values are mean ± S.E.M (*n* = 3 per group. C: control; SC: contention stress; NMS: neomaternal separation; MF: multifactorial stress; A–F: Fisher's LSD test grouping).

Regarding the oxidative stress status as evaluated in the brains of the animal models in this study, we found that *Camelina sativa* var. *Madalina* seed ME and EE have a significant overall effect on SOD-specific activity (*F* (11, 96) = 5.06, *p* = 2.39*E*^−06^) and no difference in the modulatory effect yield when administered in nonstressed mice, as comparing the seed extract solvent (*p*_ME_ = 0.22, *p*_EE_ = 0.113, as compared to the control group). Also, we observed that the greatest difference in modulatory effect was obtained while administrating the extracts in contention stress maternally separated model (*p* < 0.001). Moreover, we observed that both ME and EE significantly expressed a negative SOD activity modulation while administered to contention and multifactorial stress-exposed mice (*p*_ME_ < 0.001, *p*_EE_ < 0.001, as compared to stress positive control) and contention stress-exposed maternally separated mice (*p*_ME_ = 0.013, *p*_EE_ = 0.007, as compared to stress positive control) (Figure 5(a)).

While analysing the effect of *Camelina sativa* var. *Madalina* seed extracts on GPx-specific activity, we observed significant overall differences in GPx modulation between the studied groups (*F* (11, 96) = 2.62, *p* = 0.005) and significant differences between the ME modulatory potential when administered in nonstressed mice (*p* = 0.048) which was opposite to the effect observed when administered to the joint stress exposure mice (*p* = 0.007). The antioxidant potential of EE was observed when they were administered in the joint stress exposure mice (*p*_EE_ = 0.034) (Figure 5(b)). Moreover, it was observed that ME- and EE-treated mouse brain GPx could even exceed the positive controls levels while administered to contention stress-exposed maternally separated mice (*p*_EE_ = 0.043) and joint stress exposure mice (*p*_ME_ = 0.001). However, the greatest yielding in brain GPx activity modulation was observed for ME in the joint stress exposure mice group (*p* = 0.05), while EE was more efficient in positive modulation of brain GPx in contention stress-exposed maternally separated mice (*p* = 0.008).

Further biochemical analysis of the brain sample extracts revealed that the *Camelina sativa* var. *Madalina* effect on MDA levels was statistically significant in overall comparison (*F* (11, 96) = 17.59, *p* = 1.7749*E*^−19^], but no difference was observed between the extracts while administered to the nonstressed mice (*p*_ME_ = 0.199, *p*_EE_ = 0.261). However, significant differences between the two tested extracts were observed in the contention stress-exposed maternally separated mice (*p* = 0.015), in the contention and multifactorial stress-exposed mice (*p* < 0.001), and in the joint stress exposure mice (p < 0.001), with better yielding in the latter. In terms of efficiency, in decreasing the MDA brain levels, we observed that both ME and EE were significantly improving the lipid peroxidation process in the contention and multifactorial stress-exposed mice (*p*_ME_ < 0.001, *p*_EE_ = 0.013). Despite that, we observed that in the contention stress-exposed maternally separated mice, the EE significantly increased the lipid peroxidation process (*p* = 0.006) in the brain tissue. Also, our results suggested that the ME could exhibit a potential inflammatory effect (SC+NMS+MF+ME vs. C+ME, *p* < 0.001; SC+NMS+MF+ME vs. SC+NMS+MF, *p* < 0.001) with the greatest income in the joint stress exposure mice brains (Figure 5(c)).

Regarding total protein levels in brain tissues, when we analysed the overall effect of *Camelina sativa* extracts, we found significant differences between groups (*F* (11, 96) = 3.64, *p* = 0.00021) and significant differences between the extracts when administered to the contention and multifactorial stress mice (*p* = 0.002). In the same stress paradigm, we observed significant increases of total soluble protein brain levels following both extract administration (*p*_ME_ = 0.037, *p*_EE_ < 0.001) (Figure 5(d)).

#### 3.3.2. The Effect of *Camelina sativa* Seed Methanolic and Ethanolic Extracts on Bowel Tissue Oxidative Stress

**Figure 6:**
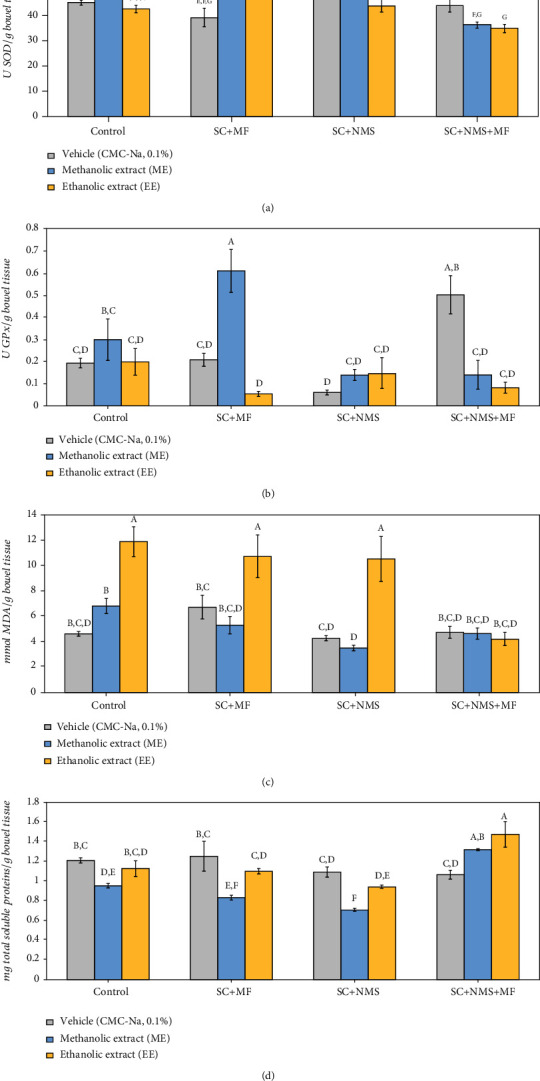
The effects of *Camelina sativa* var. *Madalina* methanolic (ME) and ethanolic (EE) extracts on bowel tissue oxidative stress markers in complex irritable bowel syndrome mouse model. (a) SOD-specific activity (U/g bowel tissue). (b) GPx-specific activity (U/g bowel tissue). (c) MDA brain content (mmol MDA/g bowel tissue). (d) Total soluble protein bowel tissue content (mg proteins/g bowel tissue). The values are mean ± S.E.M (*n* = 3 per group; C: control; SC: contention stress; NMS: neomaternal separation; MF: multifactorial stress; A–G: Fisher's LSD test grouping).

In this study, we also evaluated the oxidative stress changes occurring in the bowel tissues after the ME and EE administration in complex irritable bowel syndrome mouse models. In this way, our results showed that the overall effect of the extracts on bowel tissue SOD-specific activity was significant between groups (*F* (11, 96) = 5.57, *p* = 5.1*E*^−07^). Also, as comparing the differences in what concerns the effect of the two extracts, we observed no statistically significant influence on bowel tissue SOD activity when administered to nonstressed mice, as compared to double negative control (*p*_ME_ = 0.385, *p*_EE_ = 0.511). Despite this aspect, our results showed significant differences in enzymatic modulatory potential of the extracts when administered in the contention stress-exposed maternally separated model (*p* = 0.014) and also in the contention and multifactorial stress-exposed model (*p* = 0.027) with best modulatory yield in the latter model (*p*_ME_ < 0.001, *p*_EE_ = 0.027). In this context, we observed that the addition of supplementary stress exposure does not necessarily have a cumulative effect, as the bowel tissue SOD activity in the joint stress exposure mice reported a lower yet significant yield after both ME and EE administration (*p*_ME_ = 0.047, *p*_EE_ = 0.02, as compared to corresponding stress positive control) (Figure 6(a)).

Furthermore, the antioxidant potential of *Camelina sativa* var. *Madalina* seed extracts on the bowel tissue oxidative stress was also observed in the way ME and EE modulated the GPx-specific activity in the mentioned tissue (*F* (11, 96) = 4.80, *p* = 5.36*E*^−06^). Yet no difference between modulatory effects of the two extracts was shown for the bowel GPx-specific activity when administered to nonstressed mice (*p*_ME_ = 0.377, *p*_EE_ = 0.972), we observed that in the contention and multifactorial stress-exposed mice bowel tissues, the GPx-specific activity was significantly decreased after EE administration, as compared to ME administration (*p* = 0.027). Significant GPx modulatory effect in the bowel tissues was also observed for the ME (*p* = 0.002) and EE (*p* < 0.001) administration in joint stress-exposed mice. Meanwhile, the lowest GPx modulatory yielding in the bowel tissues was obtained when the extract was administered in the contention stress-exposed maternally separated mice (*p* < 0.001) (Figure 6(b)).

Overall significant differences in terms of lipid peroxidation intensity in the bowel tissues of the studied animals following ME and EE administration (*F* (11, 96) = 9.66, *p* = 7.37*E*^−12^) and EE significant potential to induce the MDA level increase when administered to nonstressed mice (*p* < 0.001) were observed. Also, we observed that in what concerns the lipid peroxidation modulatory effect, ME and EE are significantly different when administered to nonstressed mice (*p* < 0.001). Similarly, we observed that both ME and EE could exhibit prooxidant effects in the bowel tissue, as evaluated by the MDA levels, in the contention and multifactorial stress-exposed (*p*_ME_ = 0.005) and contention stress-exposed maternally separated models (*p*_EE_ < 0.001) (Figure 6(c)). However, the best yielding in lipid peroxidation inhibition was observed when EE was administered to the joint stress exposure mice (*p* < 0.001, as compared to the other EE groups).

Regarding total protein levels in bowel tissues, overall significant differences were observed (*F* (11, 96) = 7.80, *p* = 8.03*E*^−10^) and also we observed the significant potential of ME to decrease the bowel tissue total soluble protein levels in nonstressed mice, as compared to double negative control (*p* = 0.02). A similar effect was observed for the contention stress-exposed maternally separated mice (*p* = 0.022) and contention and multifactorial stress-exposed models (*p* = 0.015) which received EE, as compared to ME administration (Figure 6(d)).

### 3.4. Correlation Analysis

**Figure 7:**
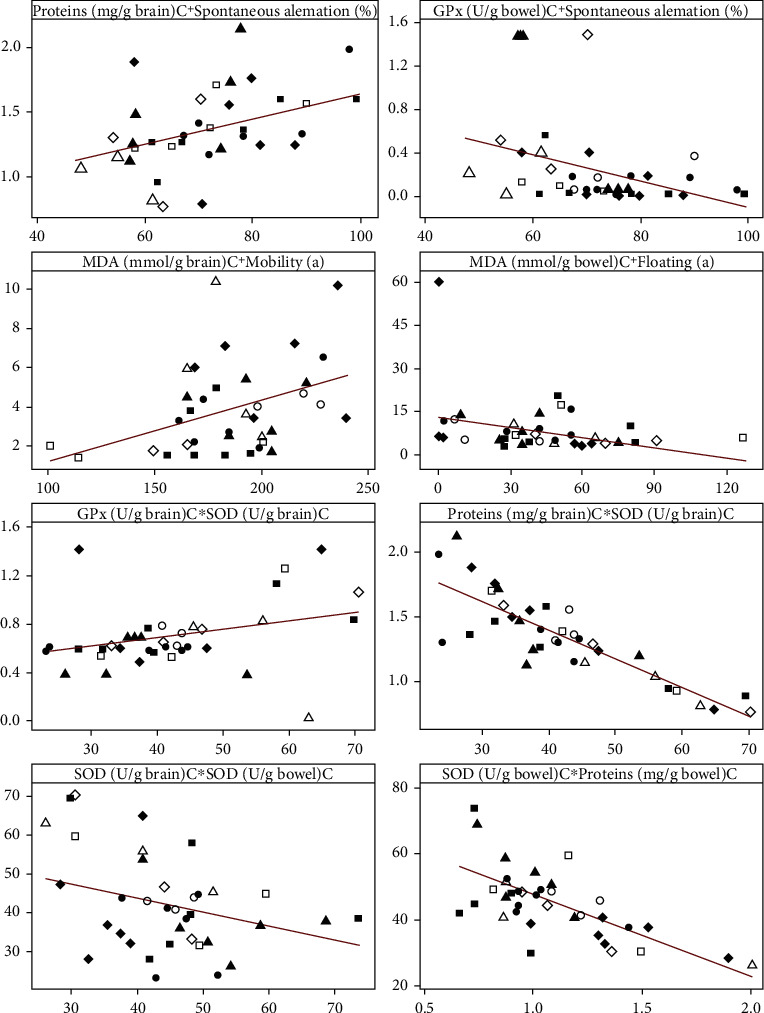
Statistical Pearson's linear correlation analysis of the behavioural parameters and oxidative stress markers (*n* = 36). The groups are marked with unique symbols: ○—control, *Δ*—SC+MF, □—SC+NMS, and ◊—SC+MF+NMS, which all received vehicle solution, whereas the black symbols (●, ▲, ■, and ♦, respectively) are for methanolic extract receiving groups and grey symbols (grey circle, grey triangle, grey square, and grey diamond●, ▲, ■, and ♦, respectively) are for the ethanolic receiving groups.

During the linear correlation analysis, we observed that some of the behavioural parameters (spontaneous alternation percentage in Y maze/open arms time and closed arms entries in elevated plus maze test/mobility and floating in forced swimming test) and the main oxidative stress markers from the brain (MDA, total proteins, and GPx) and from bowel tissues (MDA, total proteins, GPx, and SOD) could be statistically correlated. In this way, we observed the following significant correlations: total proteins (the brain) vs. spontaneous alternation (*n* = 36, *r* = 0.392, *p* = 0.005), total proteins (the brain) vs. closed arms entries (*n* = 36, *r* = 0.291, *p* = 0.042), total proteins (bowel) vs. open arm time (*n* = 36, *r* = 0.321, *p* = 0.024), total proteins (the brain) vs. the SOD brain (*n* = 36, *r* = −0.843, *p* = 0), total proteins (bowel) vs. SOD (bowel) (*n* = 36, *r* = −0.711, *p* = 0), GPx (bowel) vs. spontaneous alternation (*n* = 36, *r* = −0.353, *p* = 0.013), GPx (the brain) vs. SOD (the brain) (*n* = 36, *r* = 0.287, *p* = 0.045), SOD (bowel) vs. SOD (the brain) (*n* = 36, *r* = −0.358, *p* = 0.012), MDA (the brain) vs. mobility (*n* = 36, *r* = 0.386, *p* = 0.006), and MDA (bowel) vs. floating (*n* = 36, *r* = −0.345, *p* = 0.015) (Figure 7).

## 4. Discussion

In this study, we characterized the composition and evaluated the effects of two alcoholic extracts from *Camelina sativa* var. *Madalina* defatted seeds harvested in Romania. Also, we previously reported the effect of the *Camelina sativa* oil obtained from seeds harvested in the same year on the behavioural manifestations and oxidative stress parameters in another mouse model of irritable bowel syndrome [39].

The chemical profile of the methanolic and ethanolic extracts characterized in this study suggested that the major compound is sinapine (sinapoylcholine). The spectral peak for sinapine was obtained at m/z 310 corresponding to the protonated molecule recorded in the ESI positive mode, and the maximum of absorbance at *λ* = 331 nm was made based on earlier reported data [48–50]. Also, our results showed that the sinapine content in the characterized extracts was in good agreement with the earlier reported studies for nonoily products obtained from *Camelina* sp. (51.572% and 31.681% in methanolic and ethanolic extracts, respectively). The antioxidant, anti-inflammatory, and neuroprotective effects of sinapine were previously demonstrated as it also was described to be present in canola seed cakes [50–52] and canola meals [48, 53]. Moreover, several antioxidant flavonoid glycoside derivatives [54], which were revealed in the studied extracts by the chemical characterization, quercetin-2^″^-O-apiosyl-3-O-rutinoside and rutoside, showed a broad absorption band at 300-325 nm similar to the other flavonoid derivatives, as rutoside identification also used external standard [51]. However, some compounds from both the studied extracts remained unidentified, as could be seen in Table 1.

In this context, the present study focused on the potentially antioxidant and neuropharmacological active profile of *Camelina sativa* var. *Madalina* seed methanolic and ethanolic extracts on a stress-induced animal model of irritable bowel syndrome. The main behavioural effects exhibited by the alcoholic extracts of *Camelina sativa* seeds were memory enhancement and affective spectrum-like symptom alleviation. On the other hand, it was observed that the studied extracts could have a significant potential in oxidative status improvement in both the brain and bowel tissues in correlation to the observed behavioural effects.

Regarding the cognitive performance, the extracts of *Camelina sativa* var. *Madalina* seeds showed a beneficial effect on short-term memory in all the stress-exposed groups. Our previous findings showed that stress-exposed animals in known functional gastrointestinal syndrome modulation could lead to negative effects on the cognitive performance (short-term memory loss, affective spectrum-like symptoms occurrence, and social impairment) [55]. In this context, we observed that significant improvement of the short-term memory impairments following the heterotypical stress exposure could be obtained when the ethanolic extract was administered as a chronic treatment. By comparison, the methanolic extract was showed to be more effective in the joint stress exposure model given that the cumulative effect of the three stressors were hypothesized. However the cognitive impairment in stress-based IBS animal models was rather common; the human studies reported conflicting results regarding the occurrence of cognitive deficits in IBS patients [56, 57]. Similarly, several conflicting evidence about the relationship between stress and severity of IBS suggested that the relationship between stress and IBS symptomatology could be reciprocal, yet not causal [58], while other studies showed that stressful life events could induce and exacerbate abdominal pain and distension in a number of IBS patients [59].

There are, on the other hand, several reports on human cognitive dysfunction in IBS patients which could be a consequence of mood disorders resulting from the perceived psychological burden of the IBS gastrointestinal symptomatology [60, 61]. In this way, we also observed that the *Camelina sativa* var. *Madalina* seed alcoholic extracts possessed significant anxiolytic and antidepressant potential, as it could be described by the results obtained in the behavioural assessment of the animals. According to the studies which showed that anxiety-like and depressive-like tendencies are commonly seen in IBS patients [57], we focused on this aspect when evaluating the extracts' effects. Thus, noticeable improvement in anxiety-like behaviour was observed in all the stress exposure-based models, as it could be seen in the open arms time increase and also the anxiolytic grooming behaviour frequency following the administration of *Camelina sativa* seed extracts. However, we observed that the ethanolic extract was more efficient in this aspect suggesting that the differences in chemical composition could motivate these effects. The apparently less active methanolic extract also produced an improvement in the evaluated anxiety-like behaviour occurrence and intensity with a greater prevalence in the maternal separation model. Based on the difference between the neurological and molecular mechanisms underlying the behavioural impairments resulting in the different stress exposure paradigms and on the chemical profile differences between the extracts, we could suggest that the extracts composition could play an important role in the determination of the expected behavioural effects.

Furthermore, based on our previous experience on stress-conditioned depressive-like behaviour occurrence in chronic unpredictable mild stress rat models [13], our results suggested that both the extracts possess a significant inhibitory effect regarding the occurrence and the intensity of the depressive-like behaviours, such as the mobility time (in the forced swimming test). Based on the chemical characterization, it could be suggested that the extracts' flavonoids and phenolic acids' high content could be responsible on the antidepressant effect [62]. In addition, a valuable argument in this hypothesis, it was shown that the neurotrophic factors are playing pivotal roles in the survival, growth, and function of neurons [63] and that the flavonoids are implicated in neurotrophic factor modulation [64]. In this way, a recent study showed that this class of compounds was highly active in inducing the synthesis and secretion of neurotrophic factors, including nerve growth factor, glial-derived neurotrophic factor, and brain-derived neurotrophic factor. In this way, together with our results, it was suggested that flavonoids are an important player in the brain protection against neurodegenerative diseases [65] and also affective disorders by enhancing memory, learning, and cognitive functions [66].

In this study, the antioxidant potential of the two obtained alcoholic extracts was evaluated. In this way, the results suggested that the extract administration lead to the improvement of the antioxidant system in both the brain and bowel tissues. However, several differences were noted based on the technique used in extract accession. In this way, methanol was widely considered more efficient in plant active compound extraction due to its properties of lower molecular weight polyphenol separation [67]. Despite that, it is generally known that methanol is not safe for human consumption, unlike ethanol which is also an excellent polyphenol separation vehicle [68]. Considering these aspects, and also our results on the chemical profile of the two studied seed extracts, it could be suggested that both methanol and ethanol could have different impacts on active compound extraction yields, phytochemical constituents, and antioxidants. In this way, our results showed that better antioxidant potential was observed for the ethanolic extract, as it was also suggested by Do et al. in a study regarding the antioxidant activity of *Limnophila aromatica* ethanolic extract by contrast to methanolic, watery, and other solvents [69] and by Mondal et al. [70] in their study on the analgesic, muscle relaxant, locomotor, antiepileptic, and antidepressant effects of *Abrus precarious* ethanolic extract. On the other hand, Truong et al. argued about the anti-inflammatory effect of *Severinia buxifolia* extract in methanol, as compared to other solvents, and found that it provided the highest extraction yield of the phenolic, alkaloid, and flavonoid phases [67]. However, the study of Chauhan et al. regarding the effect of *Centella asiatica* extract in a rat model of diabetes showed that the efficiency of the methanolic and ethanolic extracts was comparable in regarding the potential to lower the blood glucose levels [71].

Thus, as also our results showed, it seems that the differences between methanol and ethanol potential as a solvent used in active compounds extraction are reflected by their effects on the antioxidant potential. The general finding our study provided is that regardless the solvent used to obtain the seed extracts, *Camelina sativa* var. *Madalina* seeds extracts possess a significant antioxidant potential in the studied stress-based IBS models. In this way, our results showed the inhibitory effect of the extract administration on the brain and bowel SOD activities which suggested that, together with the promotory effect on the GPx activity, the active compounds which can be found in the defatted seed extracts could successfully modulate antioxidant enzyme activity. In this way, the better yield in enzymatic modulation was shown by the ethanolic extract, but the significant ranges on the extracts' effects between the stress types applied to the animals suggested that the mechanisms underlying the modulation could be different. Thus, further analysis is needed to determine the impact of the active compound content on the modulatory effect and also the impact the solvent could exhibit, since ethanol was previously documented as a potent antioxidant enzyme modulator [72]. However, other studies show significant effects of other flavonoid compounds on the antioxidant enzyme activity, such as withanolide, the major component of *Withania somnifera* extract when administered to an epileptic rat model [73].

Moreover, regarding the effect of *Camelina sativa* var. *Madalina* extracts on the brain and bowel tissue biochemical features, we observed that both methanolic and ethanolic extracts possessed the ability to modulate lipid peroxidation, while methanolic extract was more efficient. In this way, it could be mentioned that the methanolic extract was able to significantly decrease lipid peroxidation (as suggested by the decrease of the MDA content) in the tissues of the animals which were exposed to heterotypical stress factors. Furthermore, similarly to its effect on the antioxidant enzymes, the ethanolic extract showed a significant potential to promote lipid peroxidation in the bowel tissue in three out of the four animal groups in our study. This effect could be argued against the ethanol properties and also against the sinepine bitterness and astringency which were previously compared to caffeine [74].

However, some limitations of this study must be discussed. Thus, mainly due to the small number of tested animals and the absence of positive control drug treatments, this preliminary study could only address some descriptive and correlative aspects underlying the complex effects of *Camelina sativa* var. *Madalina* seed extracts. However, phenolic compounds can prevent neurodegenerative diseases and memory deficits that depend on oxidative stress. Oral administration and supplementation of diets with phenolic extracts improve the memory of mice and prevent memory deficit associated with age-related diseases.

It is well known that the extracts rich in bioactive compounds (e.g., phenolic compounds such as quercetin and its derivatives) demonstrated to possess antioxidant capacity decrease the oxidative stress parameters [75–82] and acetylcholine enzyme (AChE) inhibitory activities that are regarded as a suitable strategy for preventing memory deficits caused by neurodegenerative damages. The cholinergic pathways are implicated in the pathophysiology of learning and memory disorders as was earlier reported [83, 84].

Our results showed that the administration of the alcoholic extracts of Camelina sativa seeds presented a mixture of bioactive compounds with antioxidant potential that lead to a decrease of the superoxide dismutase activity and increase of the glutathione peroxidase activity. The mechanism probably involved the cholinergic pathways due to the synergic activities of flavonoid (quercetin derivatives) and glycoside derivatives. Thus, the future approaches in this context are to improve the experimental design by using common treatments which could point to specific pathways of action and by using the extract components as sole treatments or combined administrations in order to establish their independent or synergic effects, as well as their potential to pass through the blood-brain barrier.

Considering that few studies reported the *Camelina sativa* seed extract potential, we provided additional evidence on the chemical description of the methanolic and ethanolic extracts and their effect on the behavioural and biochemical aspects of several stress-based irritable bowel syndrome combined models. Thus, the omega-3 fatty acids, polyphenols, and natural antioxidants [85] rich seed extracts were suggested to possess neurophysiological and antioxidant potential, as also other studies an important role in reversing oxidative stress and improving mood and cognitive psychopathologies [86, 87]. Consequently, our results showed that methanolic and ethanolic *Camelina sativa* var. *Madalina* seed extracts could be used in alleviating behavioural and oxidative stress burden in a stress-based functional gastrointestinal disorder animal model.

## 5. Conclusions

This study showed the beneficial effects of *Camelina sativa* seed methanolic and ethanolic extracts on the behaviour and brain and bowel tissue oxidative stress status of stress exposure-based irritable bowel syndrome mouse models. Despite the slight differences in the chemical composition of the methanolic and ethanolic extracts, the results suggested that the *Camelina sativa* extracts could reverse the short-term memory impairments caused by stress exposure and also could decrease the intensity and frequency of the anxiety and depressive-like behaviours observed in the stress-exposed animal models of irritable bowel syndrome. Furthermore, the *Camelina sativa* extracts showed a significant effect on the oxidative stress markers in the brain and bowel tissues of the studied animal model by decreasing the superoxide dismutase activity and increasing the glutathione peroxidase activity. However, the results suggested that the extracts could also increase lipid peroxidation in bowel tissues. In this way, this study provides additional evidence that the administration of *Camelina sativa* seed alcoholic extracts could improve cognitive performances and mood and exhibit antioxidant capacity in both the brain and bowel tissues.

## Figures and Tables

**Table 1:** Identification of major compounds in alcoholic *Camelina sativa* var. *Madalina* seed extracts by HPLC-DAD-MS (ME: methanolic extract; EE: ethanolic extract).

Peak	Compound	RT (min)	*λ*_max_	[M]^−^ [M]^+^ (m/z)	MS fragment ions (m/z)	Relative amount (%) ME	Relative amount (%) EE
1	NI	13.33	266	—	—	0.674	0.420
				520	292, 251, 201, 172		
	NI	13.87	201	565	505	0	0.802
				453	428, 292, 251, 201		
2	Glucoarabin	13.93	325	506		2.071	1.556
				428^∗^	406, 349, 311, 261, 216		
3	Glucocamelinin	14.31	255, 353	520		11.265	14.999
				442^∗^	393, 292, 251		
4	Quercetin-2^″^-O-apiosyl-3-O-rutinoside	14.71	221, 302	741	520, 395	1.888	1.756
				743^∗^	476, 442, 292, 230, 216, 172		
5	NI	14.988	255, 353	—	—	24.601	15.664
				720^∗^	382, 292, 253, 230, 205, 172		
6	Rutoside (quercetin-3-O-rutinoside)	15.39	239, 325	610	534	1.984	3.735
				611^∗^	456, 440, 294, 244, 227, 172		
7	NI	16.037	238, 328	456	294, 244, 230, 216	2.924	0.507
8	Sinapine	16.26	238, 331	—	—	51.572	31.681
				310	251, 201, 199, 172		
9	Gluconesliapaniculatin	18.117	214, 326	—	—	3.021	3.845
				534	310, 292, 251, 201, 172, 132		
10	NI	20.16	236, 325	—	—	0	24.21
				517	292, 251, 238, 201, 172, 132		

^∗^NI: nonidentified.

## Data Availability

The scientific data used to support the findings of this study are available upon request.
